# Salvianolic acids from antithrombotic Traditional Chinese Medicine Danshen are antagonists of human P2Y_1_ and P2Y_12_ receptors

**DOI:** 10.1038/s41598-018-26577-0

**Published:** 2018-05-24

**Authors:** Xuyang Liu, Zhan-Guo Gao, Yiran Wu, Raymond C. Stevens, Kenneth A. Jacobson, Suwen Zhao

**Affiliations:** 1 iHuman Institute, ShanghaiTech University, Shanghai, 201210 China; 2 School of Life Science and Technology, ShanghaiTech University, Shanghai, 201210 China; 3 Key Laboratory of Computational Biology, CAS-MPG Partner Institute for Computational Biology, Shanghai Institutes for Biological Sciences, Chinese Academy of Sciences, Shanghai, 20031 China; 4 University of Chinese Academy of Sciences, No. 19A, Yuquan Road, Beijing, 100049 China; 5 Molecular Recognition Section, Laboratory of Bioorganic Chemistry, National Institute of Diabetes and Digestive and Kidney Diseases, National Institutes of Health, Bethesda, Maryland 20892 USA

## Abstract

Many hemorheologic Traditional Chinese Medicines (TCMs) that are widely-used clinically lack molecular mechanisms of action. We hypothesized that some of the active components of hemorheologic TCMs may function through targeting prothrombotic P2Y_1_ and/or P2Y_12_ receptors. The interactions between 253 antithrombotic compounds from TCM and these two G protein-coupled P2Y receptors were evaluated using virtual screening. Eleven highly ranked hits were further tested in radioligand binding and functional assays. Among these compounds, salvianolic acid A and C antagonized the activity of both P2Y_1_ and P2Y_12_ receptors in the low µM range, while salvianolic acid B antagonized the P2Y_12_ receptor. These three salvianolic acids are the major active components of the broadly-used hemorheologic TCM Danshen (*Salvia militorrhiza*), the antithrombotic molecular mechanisms of which were largely unknown. Thus, the combination of virtual screening and experimental validation identified potential mechanisms of action of multicomponent drugs that are already employed clinically.

## Introduction

Many hemorheologic agents in Traditional Chinese Medicine (TCM) are still widely used, including Chuanqiong (*Ligusticum wallichii*)^1^, Danshen (*Salvia militorrhiza*)^2^, and Honghua (*Carthamus tinctorius*)^3^. These hemorheologic agents are highly efficacious, although they often manifest side effects. A common strategy for developing safer drugs includes identifying and extracting the active components of TCMs. However, the molecular mechanisms including targets of their active components remain unknown in most cases. For example, Danshen depside salt, containing only several water-soluble components of Danshen^4,5^, has fewer side effects and is one of the common hemorheologic treatments used in China^4,6,7^. The principal water-soluble component of Danshen is salvianolic acid B (SAB), however, several other salvianolic acid variations, including salvianolic acid A (SAA) and salvianolic acid C (SAC), are also major components^5^. While SAA, SAB and SAC block adenosine 5′-diphosphate (ADP)-induced platelet aggregation^8–10^ more potently than other known inducers, such as thrombin and collagen^11^, the molecular mechanism of the antithrombotic action of these salvianolic acids, including their target receptors, remains largely unknown^11^. A recent study using atomic-force microscopy concluded that SAB can bind to the purinergic G protein-coupled receptor (GPCR) P2Y_12_; however, only a single dose of 280 μM was tested^10^.

We hypothesized that the active components of hemorheologic TCMs may function through targeting the purinergic GPCRs P2Y_1_ and/or P2Y_12_ due to their critical roles in platelet aggregation^12^. Both P2Y receptors can be activated by the same endogenous ligand, ADP^13^. Coactivation of the two receptors is essential for ADP-induced platelet aggregation^12^. Specifically, activation of the G_q_-coupled P2Y_1_ receptor leads to intracellular calcium mobilization, platelet shape change and formation of small, reversible aggregates. Activation of the G_i_-coupled P2Y_12_ receptor leads to a decrease in the level of cyclic adenosine 3′,5′-monophosphate (cAMP) that drives the reversible platelet aggregates to an irreversible state^14,15^. As a result, both P2Y receptors are antithrombotic drug targets; inhibition of both receptors has a synergistic effect^16–18^. Several antithrombotic drugs, such as clopidogrel, prasugrel^19^ and ticagrelor^20^, antagonize the P2Y_12_ receptor. However, no drugs on market targets the P2Y_1_ receptor. Since the structures of the P2Y_1_ and P2Y_12_ receptors were solved recently using X-ray crystallography^21–23^, we were able to take a structure-based virtual screening approach to predict ligands of the P2Y_1_ and P2Y_12_ receptors from antithrombotic components of TCMs.

## Results

### Eleven hits for P2Y receptors were found by ensemble docking

**Figure 1:**
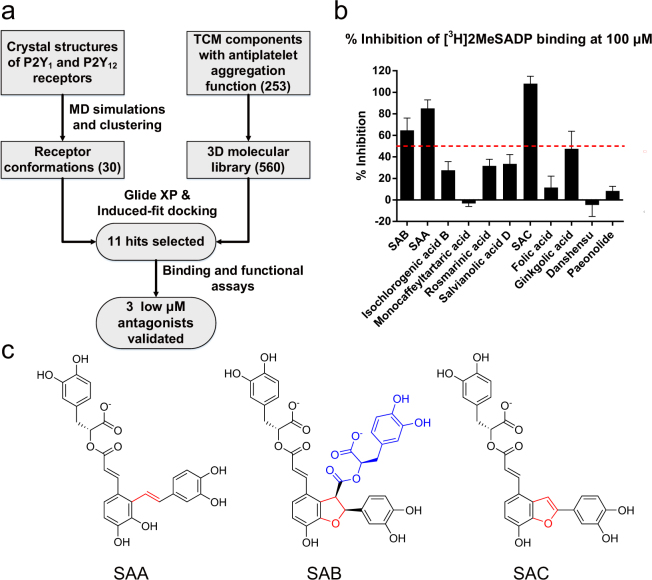
Summary of structure-based TCM ligand discovery strategy for P2Y_1_ and P2Y_12_ receptors. (**a**) Flowchart of ligand discovery for P2Y_1_ and P2Y_12_ receptors from TCM components with antiplatelet aggregation function. (**b**) Percentage inhibition of the binding of [^3^H]2MeSADP to the P2Y_12_ receptor by 11 hits (at 100 μM) selected from virtual screening. (**c**) 2D structures of three validated hits, SAA, SAB and SAC. The shared parts in the three compounds are in black, the different linker regions are in red, and the unique extra moiety in SAB is in blue.

Ensemble docking, a method that has recently been widely used for GPCR ligand discovery^24–26^ since GPCRs tend to have flexible binding pockets^21,27,28^, was adopted for this study. In order to get multiple receptor conformations, 100 ns MD simulations were performed starting with the crystal structures of the P2Y_1_ (PDB: 4XNW) and P2Y_12_ receptors (PDB: 4PXZ and 4NTJ) (Figure S1). The virtual screening library consists of 253 antiplatelet aggregatory TCM components. Following the ensemble docking, the top fifty ligands ranked by Glide XP scores were further re-ranked by Induced Fit Docking^29^ that allows residues within 5 Å of the ligand to move. Finally, eleven hits were manually picked for experimental testing in part based on their commercial availability, resulting in the identification of SAA and SAC as antagonists of the P2Y_1_ and P2Y_12_ receptors at low µM level, while SAB inhibits only the P2Y_12_ receptor (Fig. 1a).

### Three ligands have antagonist activity against P2Y_1_ and/or P2Y_12_ receptors

The eleven selected components were further examined in radioligand binding and functional assays. We initially tested the eleven compounds by measuring the inhibition of [^3^H]2-methylthioadenosine 5′-diphosphate (2MeSADP) binding at the human P2Y_12_ receptor (Fig. 1b). The three salvianolic acids from Danshen, SAA, SAB and SAC (Fig. 1c), were found to have significant inhibitory effect on P2Y_12_ receptor binding at a concentration of 100 µM. The remaining eight tested components, including salvianolic acid D (33.5 ± 8.6% inhibition), were weakly inhibiting or inactive. Another Danshen component, rosmarinic acid, was previously shown to be more potent in antagonizing thrombin-induced than ADP-induced platelet aggregation^30^. Our results confirmed that it is indeed a weak ligand of the P2Y_12_ receptor (31.6 ± 6.2% inhibition at 100 µM).

**Figure 2:**
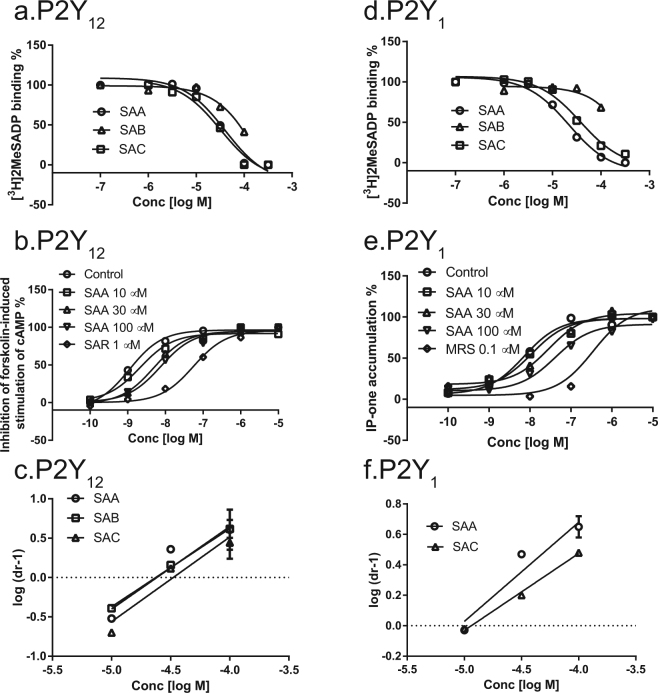
Radioligand binding assays and functional assays showed that SAA and SAC can bind and antagonize both P2Y_1_ and P2Y_12_ receptors, while SAB can bind and antagonize only the P2Y_12_ receptor. (**a**) Displacement curves of SAA, SAB and SAC against [^3^H]2MeSADP binding to the P2Y_12_ receptor. (**b**) Functional antagonism by SAA of 2MeSADP-induced inhibition of foskolin-stimulated cAMP accumulation in U2OS cells expressing the P2Y_12_ receptor. (**c**) Schild plots of the antagonism of SAA, SAB and SAC at the P2Y_12_ receptors. (**d**) Displacement curves of SAA, SAB and SAC against [^3^H]2MeSADP binding to the P2Y_1_ receptor. (**e**) 2MeSADP-induced IP-1 accumulation in U2OS cells expressing the P2Y_1_ receptor (compared to MRS2500). (**f**) Schild plots of antagonism by SAA and SAC at the P2Y_1_ receptor. Results are expressed as mean ± SEM. The K_i_ values from binding experiments and K_B_ values from Schild analyses of functional antagonism by SAA, SAB and SAC are listed in the text and are from at least three independent experiments. MRS, MRS2500; SAR, SAR216471.

The binding affinities of SAA, SAB and SAC for the P2Y_12_ receptor were determined by full inhibition curves resulting in K_i_ values of 20.3 ± 1.5, 36.4 ± 20.3, 15.6 ± 6.5 µM, respectively (Fig. 2a). The functional antagonism of these three salvianolic acids against the P2Y_12_ receptor was measured by their effect on agonist-induced inhibition of forskolin-stimulated cyclic AMP accumulation in P2Y_12_ receptor-expressing U2OS cells. For example, in a concentration-dependent manner, SAA shifted the agonist response curve to the right without affecting its maximum agonist effect (Fig. 2b). This observation is consistent with competitive antagonism, but allosteric antagonism cannot be excluded^31^. Schild analysis of the antagonism yielded K_B_ values of 24.2 ± 5.9, 33.6 ± 6.4 and 23.7 ± 4.5 µM for SAA, SAB and SAC, respectively (Fig. 2c).

Both P2Y_1_ and P2Y_12_ receptors play important roles in platelet aggregation. Therefore, we also tested the potential effects of SAA, SAB and SAC on the P2Y_1_ receptor. Initial binding results showed that both SAA and SAC produced a substantial inhibitory effect on [^3^H]2MeSADP binding to the P2Y_1_ receptor while the inhibition by SAB was significantly weaker (Fig. 2d); the concentration-dependent inhibitory curves for SAA and SAC resulted in K_i_ values of 14.7 ± 2.4 and 23.3 ± 4.5 µM, respectively. SAA and SAC antagonized 2MeSADP-induced accumulation of inositol 1-phosphate (IP-1) (Fig. 2e). K_B_ values calculated by Schild analysis for the P2Y_1_ receptor were 9.1 ± 1.5 and 15.6 ± 2.0 µM for SAA and SAC, respectively (Fig. 2f). Thus, the Schild analysis of the antagonist potencies of SAA and SAC for G_q_-coupled P2Y_1_ and G_i_-coupled P2Y_12_ receptors was consistent with their binding affinities measured in the radioligand assays.

To examine the possibility that the observed pharmacological activity might be due to colloidal aggregation of these compounds, we tested the effect of a nonionic detergent, Tween-20^32–34^, on the inhibition of the specific binding of [^3^H]2MeSADP to the P2Y_1_ receptor by the two potent salvianolic acids (SAA and SAC). We found that the inhibition curves are not rightward-shifted by Tween-20 (Figure S2). Thus, the results suggest that colloidal aggregation is not a major contributor to the inhibition of the specific binding of [^3^H]2MeSADP by salvianolic acids. Additionally, the reports that SAA and SAC are more potent at ADP-induced than at thrombin-induced platelet aggregation^8,35^ may also support a specific effect of these compounds.

### SAA, SAB and SAC may act through additional targets

We also tested if SAA, SAB and SAC are promiscuously binding compounds, due to the fact that they all have multiple phenolic groups and are thus potentially PAINS^36^. To find other possible targets of the three compounds, we evaluated their potential binding to 45 diverse receptors, transporters and ion channels (human, unless noted) using radioligand binding assays *via* the Psychoactive Drug Screening Program (PDSP) (Table S1)^37^. Among the three compounds, only SAB bound to one off-target site, i.e. the α_1B_ adrenergic receptor with a K_i_ value of 6.26 ± 1.46 µM (n = 3) (Figure S3), however following FLIPR assays showed that SAB is neither agonist nor antagonist of α_1B_ (Figure S4). No other significant affinity (<50% inhibition at 10 µM) was detected for any of the proteins listed in Table S1. Therefore, these natural products may have multiple targets *in vivo*, but do not display the indiscriminate promiscuity characteristic of PAINS compounds.

### Binding poses of SAA, SAB and SAC are consistent with their functions

**Figure 3:**
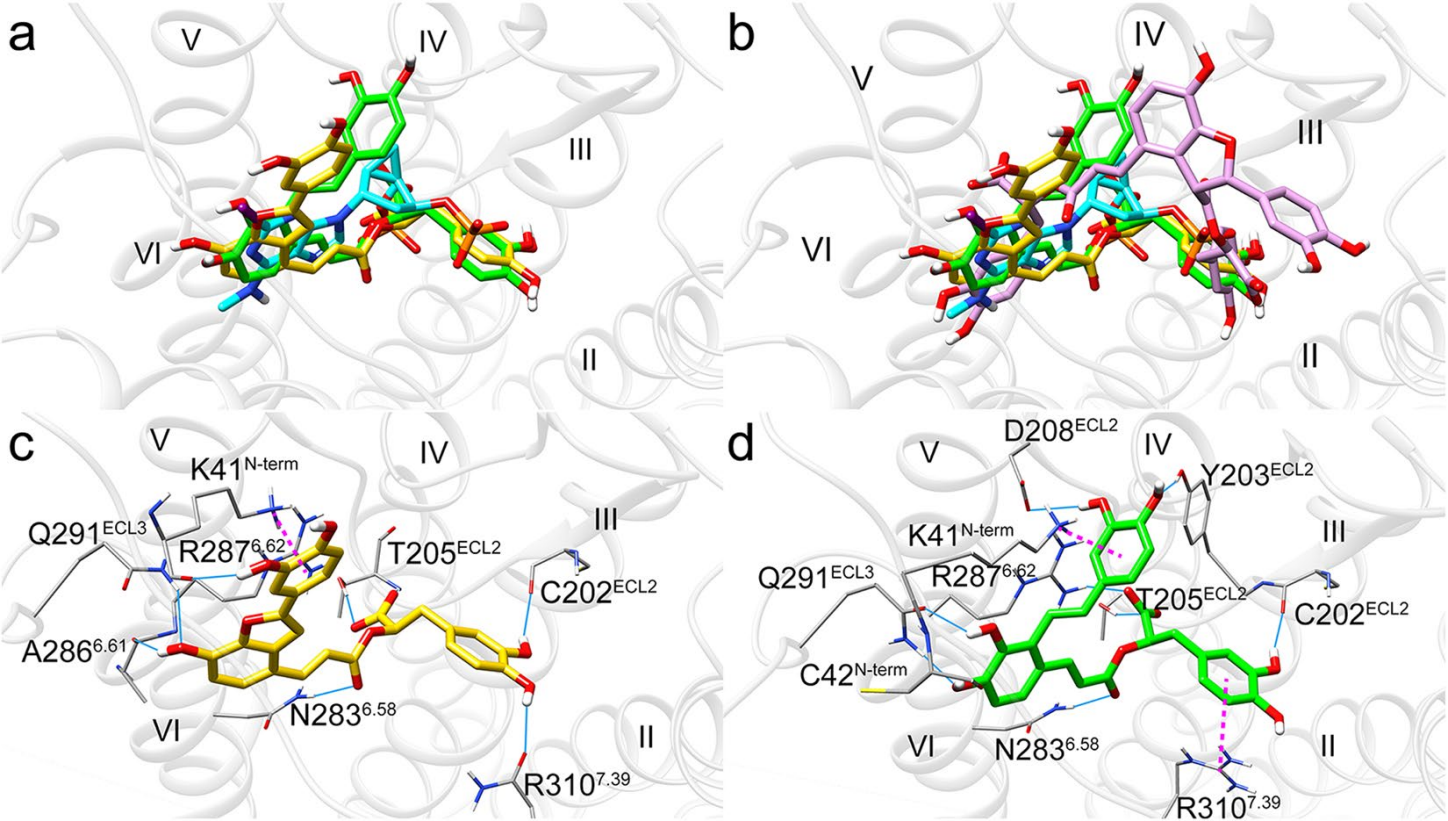
Docking poses of SAA (green), SAB (purple) and SAC (yellow) in P2Y_1_ receptor. (**a**) Comparison of the docking poses of SAA and SAC with co-crystalized antagonist MRS2500 (cyan) in P2Y_1_ receptor shows that SAA and SAC align well with MRS2500. (**b**) Comparison of the binding pose of SAB with MRS2500, SAA and SAC shows that it does not occupy the same space as MRS2500/SAA/SAC. (**c**,**d**) SAA and SAC have good interactions with key residues in the pocket of P2Y_1_ receptor. Light blue solid lines and magenta dashed lines represent hydrogen bonds and π-π/cation-π interactions, respectively.

We analyzed the binding poses of SAA, SAB and SAC in the binding sites of P2Y_1_ and P2Y_12_ receptors. Their poses were consistent with their antagonistic function against these receptors. For the P2Y_1_ receptor, the binding poses of SAA and SAC were similar to the co-crystalized antagonist (1′*R*,2′*S*,4′*S*,5′*S*)-4-(2-iodo-6-methylamino-purin-9-yl)-1-[(phosphato)-methyl]-2-(phosphato)-bicyclo[3.1.0]hexane (MRS2500) (Fig. 3), consistent with their function as P2Y_1_ antagonists. However, unlike SAA and SAC, SAB had a more external binding pose (Fig. 3b), likely due to the steric bulk of SAB, consistent with SAB having little effect on the P2Y_1_ receptor.

**Figure 4:**
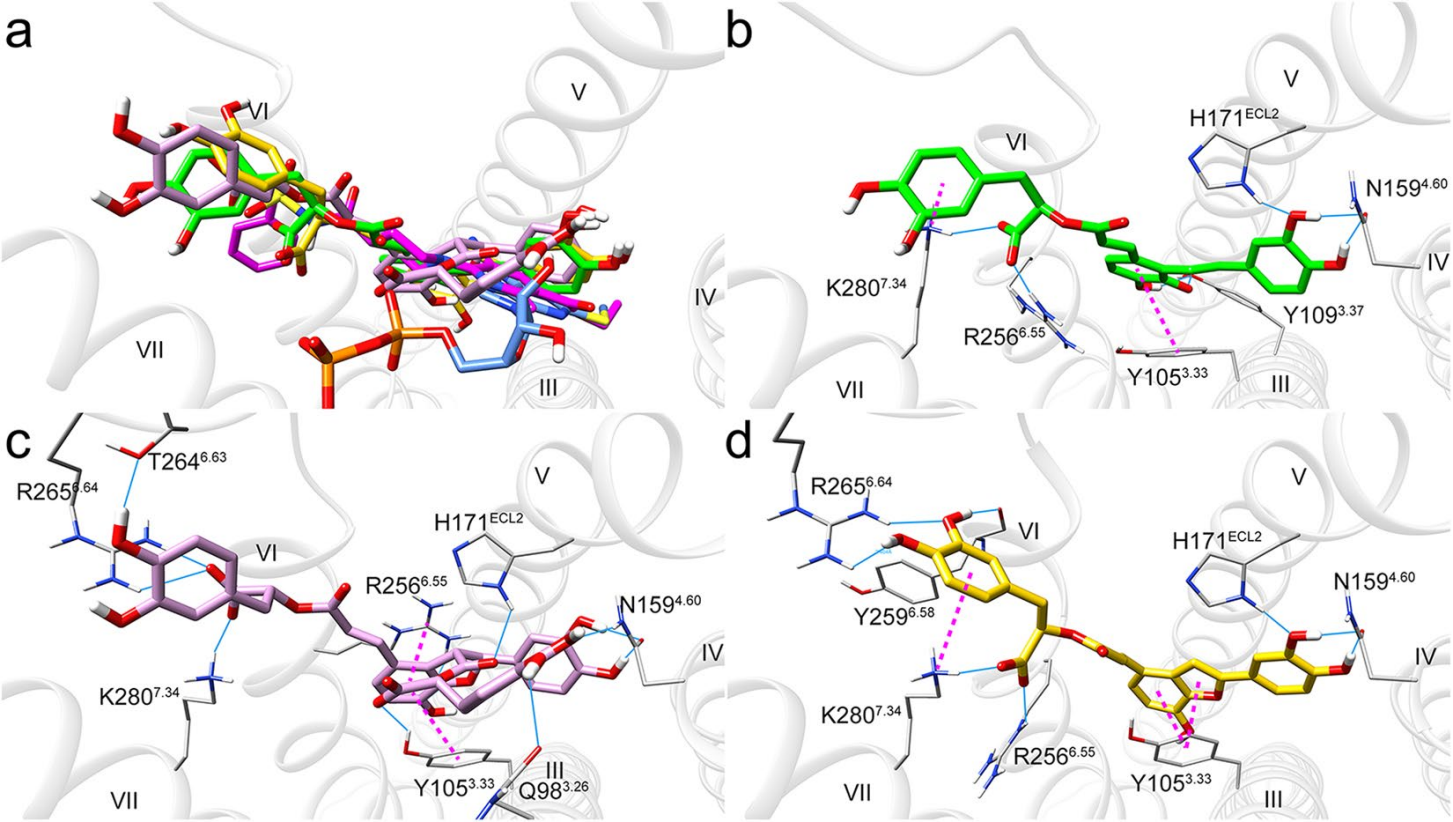
Docking poses of SAA (green), SAB (purple) and SAC (yellow) at the P2Y_12_ receptor. (**a**) Comparison of the docking poses of SAA, SAB and SAC with co-crystalized antagonist AZD1283 (magenta) and agonist 2MeSADP (blue) in P2Y_12_ receptor show that SAA, SAB and SAC can superimpose with the co-crystalized antagonist but not the agonist. (**b**–**d**) SAA, SAB and SAC bind the P2Y_12_ receptor in a similar pose and show good interactions with residues lining the pocket. Light blue solid lines and magenta dashed lines represent hydrogen bonds and π-π/cation-π interactions, respectively.

At the P2Y_12_ receptor, SAA, SAB and SAC were predicted to bind similarly, interacting with all transmembrane helices except I and II, mimicking the co-crystalized antagonist ethyl 6-(4-((benzylsulfonyl)carbamoyl)piperidin-1-yl)-5-cyano-2-methylnicotinate (AZD1283) but not the co-crystalized agonist 2MeSADP (Fig. 4). These models are consistent with the fact that all three salvianolic acids are inhibitors of the P2Y_12_ receptor. Each of the three compounds interacted with several key residues such as Tyr105^3.33^ (Ballesteros-Weinstein numbering^38^ scheme), Asn159^4.60^, His171^ECL2^, Arg256^6.55^ and Lys280^7.34^, albeit the detail interactions were slightly different. The binding poses of the three compounds showed that unpaired hydrogen bond donors and acceptors increased in the order of SAC, SAA, and SAB. It is uncertain whether these differences contributed to the small differences in their potencies.

## Discussion

A significant finding is that while SAB only antagonizes the P2Y_12_ receptor, SAA and SAC are dual inhibitors of P2Y_1_ and P2Y_12_ receptors. Simultaneous inhibition of P2Y_1_ and P2Y_12_ receptors by a combination of selective inhibitors at low concentration has already been shown to synergistically enhance antithrombotic activity, especially for high shear stress-induced platelet aggregation^5^. A combination of P2Y_1_ and P2Y_12_ receptor antagonists at low concentrations reduced platelet–platelet cohesion and mural thrombosis^12^. Although these non-purine salvianolic acids do not bind to the P2Y receptors as potently as marketed P2Y_12_ receptor antagonist drugs, they are nevertheless effective in practice. We hypothesize that the therapeutic action of these salvianolic acids, especially when administered by injection, may benefit from the synergistic antithrombotic effect of blocking both P2Y receptors. Furthermore, it is well known that nanomolar inhibitors of the P2Y_12_ receptor are associated with a significantly increased bleeding risk^19,20^. A case in point is that clinical use often requires two antithrombotic drugs. Administration of aspirin and a P2Y_12_ antagonist is recommended for all patients with a clear diagnosis of acute coronary syndrome (ACS) to balance the benefits and bleeding complications^39^. A potential advantage of the P2Y_1_ receptor antagonists as antithrombotic drugs compared to the P2Y_12_ receptor antagonists may be the controllable bleeding time^12^. Thus, the finding that multiple components from Danshen have low μM antagonistic activity against both P2Y_1_ and P2Y_12_ receptors may explain the clinical efficacy and the relatively safe use of Danshen for the prevention and treatment of ACS during the past two millennia^40^.

The antithromobotic effects of SAA, SAB and SAC both *in vitro* and *in vivo* have been previously reported^8–10,35^, although the potential mechanism mediated *via* a specific P2Y receptor has been largely unexplored. The effect of SAA was found to be more potent on ADP-induced than thrombin- or arachidonic acid-induced rat platelets aggregation both *in vivo* and *in vitro*^8^. The antiplatelet efficacy of SAB was demonstrated in ACS patients undergoing treatment with clopidogrel and aspirin^10^. SAC was found to inhibit platelet aggregation *in vitro* and *in vivo*^9,35^. In addition, although salvianolic acids are often believed to be redox-active and function as reactive oxygen species scavengers, actually their half-lives are not very short. It was reported that in rat plasma, *t*_1/2_ of SAB is about 105 min (100 mg/kg, i.v.) and 248 min (500 mg/kg, p.o.), and *t*_1/2_ of SAA is 197 min (100 mg/kg, p.o.)^11^.

SAA, SAB and SAC are polyphenolic phytochemicals, which are often thought to bind to many membrane proteins indiscriminately^36^. Indeed, it was reported SAB/SAA can bind MMP-9 (K_i_ 79.2 μM)^41^, SH2 domain of Src family kinases (IC_50_ 41–90 μM)^42^ and CD36 (IC_50_ 23.2 μM)^43^. Our study added two more members to the list: P2Y_1_ and P2Y_12_ receptors, with SAA and SAC having the highest affinities among all the determined K_i_/EC_50_/IC_50_. Our broad screening of the three compounds against 45 receptors/transporters/ion channels at PDSP found no hits, thus the three compounds pass through the orthogonal target assays suggested by the recent review on PAINS^44^. Nevertheless, the important clinical distinction is that Danshen is proven safe and efficacious in human use. Although we realize that by the current standards of pharmaceutical development it would be highly unlikely that such a drug combination would be considered now, we have shed light on the mechanism of action of a proven TCM treatment.

Finding target(s) for multicomponent-TCM is critical for understanding their mechanisms and improving the safety. Computational methods such as network-based and machine learning-based methods have been developed to predict drug-target interactions^45,46^ and synergistic drug combinations^47^. Here we used virtual screening to predict that SAA, SAB and SAC, three components of a broadly used antithrombotic TCM Danshen, bind to two known antithrombotic targets: P2Y_1_ and P2Y_12_ receptors. This prediction was validated experimentally revealing binding affinity at low μM levels and functional antagonistic activity against these two receptors. These results suggest a plausible mechanism for the antithrombotic activity of Danshen. By blocking both P2Y receptors through the three major components, Danshen exhibits a synergistic antithrombotic effect while also keeping bleeding risk tolerable. This mechanism provides a basis for further development of a dual antagonist against the P2Y_1_ and P2Y_12_ receptors as an antithrombotic agent. We showed that virtual screening and experimental validation can identify a molecular mechanism of action of multicomponent drugs that are already in clinical use.

## Methods

### Protein preparation

Agonist-bound and antagonist-bound structures of human P2Y_12_ receptor (PDB: 4PXZ, 4NTJ) and an antagonist-bound structure of human P2Y_1_ receptor (PDB: 4XNW) were prepared for molecular dynamics simulation. Fusion protein, water molecules, ions, solvent molecules and ligand were removed from the three structures. Then the structures were processed using the protein preparation wizard in Schrodinger Suite 2015–4^48^. During the process, hydrogens, disulfide bonds, missing side chains and loops were appropriately included; N-terminal and C-terminal ends were capped, and protonation states were assigned at pH 7.0 by Protassign; and a minimization with 0.3 Å RMSD constraints on heavy atoms was performed.

### Molecular dynamics simulations

Molecular dynamics simulations were performed using GROMACS 5.0.6^49^. The three prepared structures of P2Y_1_ and P2Y_12_ receptors were embedded into a POPC (1-palmytoil-2-oleoyl-sn-glycero-3-phosphatidylcholine) lipid bilayer, respectively. Membrane Builder^50^ of CHARMM-GUI^51^ was used to build the protein/membrane complex systems. Each system consisted of a receptor, a lipid bilayer of ~120 POPC molecules, ~10700 TIP3P water molecules and 0.15 M NaCl with respective counter ions. Nearly 50,000 atoms in total were contained in a periodic box ~75 × 75 × 115 Å^3^. Van der Waals and short-range electrostatic interactions were cut off at 10 Å. Equilibration was performed using a NPT run at 1 atm and 310 K for 10 ns with a time step of 1 fs. Molecular dynamics simulations were performed in the NPT ensemble for 100 ns using standard CHARMM 36 force field^51^ at 1 atm and 310 K with a time step of 2 fs.

### MD trajectories clustering

The MD simulation trajectories of the three P2Y receptor systems were used to examine their potential small molecule interactions. The ligand binding site was defined by protein residues within 5 Å of the cocrystalized ligand in each X-ray structure. Based on the root-mean-square deviation (RMSD) of the selected residues, receptor snapshots taken every 1 ps in the simulation trajectory were clustered for each target site using the g_cluster tool in GROMACS package. The gromos clustering algorithm was applied. 262, 304 and 554 clusters were generated from the MD trajectories of 4XNW, 4PXZ and 4NTJ, respectively, with a 1.0 Å RMSD cutoff. The top 10 populated clusters from each trajectory were selected for ensemble docking.

### Building a library of TCM components with antiplatelet aggregation function

A library of 253 TCM components with antiplatelet aggregation function was built. These compounds were filtered from the Traditional Chinese Medicine Database^52^ and an in-house TCM component library by using the keyword “antiplatelet aggregation”. Unspecified chiral centers were manually checked and assigned if available in literature. LigPrep was used to generate 3D structures of the ligands. During the process an OPLS3 force field^53^ was used for geometry optimization, pH 7.0 ± 2.0 was used to generate possible protonation states and the option “retain specified chiralities (vary other chiralities)” was used to generate stereoisomers. In total 560 compounds were generated through LigPrep processing.

### Ensemble Docking

Glide XP^48,54–56^ was used to dock the antiplatelet aggregation library into the 30 conformations generated from clustered MD trajectories. Docking boxes were defined by the residues in the ligand binding site. Default parameters of Glide XP were used except for the inner and outer box lengths, which were set to 14.0 Å and 28.2 Å, respectively. MM-GBSA was used to optimize ligand conformation and rescore relative binding affinity. Ligand and receptor residues within 5 Å of the ligand were allowed to move in MM-GBSA calculation. Fifty top-scored ligands were selected from all docking results. Then Induced-Fit Docking (IFD)^29,57–59^ was further applied to optimize the binding poses and binding score. Finally, 11 antiaggregatory compounds were manually selected based on their docking scores and poses for experimental validation.

### Membrane preparation and binding assays

U2Os cells expressing the P2Y_1_ or P2Y_12_ receptors were cultured in DMEM supplemented with 10% fetal bovine serum, 100 Units/ml penicillin, 100 µg/ml streptomycin and 2 µmol/ml glutamine. After reaching confluence, cells were detached from plates by scraping into PBS and centrifuged at 250 *g* for 5 min. The resulting pellets were re-suspended in ice-cold Tris-HCl buffer (50 mM, pH 7.4) and homogenized. After homogenization and suspension, cells were centrifuged at 1000 *g* for 10 min and the pellet was discarded. The suspension was re-centrifuged at 20,000 *g* for 60 min at 4 °C. The pellets were re-suspended, split into aliquots and stored at −80 °C. The protein concentration was measured using the Bradford assay. For P2Y_1_ and P2Y_12_ receptor binding assays, saturation curves were measured by addition of various concentrations of 50 µl [^3^H]2MeSADP (specific activity, 7.5 Ci/mmol; Movarek Biochemicals, Brea, CA) and 100 μl of membrane suspension to a total of 200 μl of total assay volume. Non-specific binding was determined using 10 µM MRS2500 (Tocris; St. Louis, MO) for the P2Y_1_ receptor and 10 μM SAR216471 (Tocris; St. Louis, MO) for the P2Y_12_ receptor. For the displacement assay, various concentrations of inhibitors were incubated with [^3^H]2MeSADP (1.0 nM for P2Y_1_; 0.4 nM for P2Y_12_) and membrane preparations (20 µg/sample) at 25 °C for 60 min. The separation of bound from free radioligand was accomplished by rapid vacuum filtration of the incubation mixture over GF/B filter using a Brandel cell harvester (Brandel Inc., Gaithersburg, MD). Filters were washed two times with 3 ml of ice-cold Tris-HCl buffer, pH 7.5. Radioactivity on the filters was quantified using a Tri-carb Liquid Scintillation Counter (PerkinElmer Life and Analytical Sciences). 2MeSADP was purchased from Sigma (St. Louis, MO). All other materials were from standard commercial sources and of analytical grade. For radioligand binding, calculation of apparent binding affinities, *K*_i_ = IC_50_/(1 + [radioligand]/*K*_d_), was performed using the nonlinear iterative curve-fitting program of Prism (GraphPad Software Inc., San Diego, CA).

### Functional assays

#### IP-one assay

Inositol 1-phosphate (IP-1) was measured using the IP-One Tb HTRF kit (Cisbio Bioassays, Bedford, MA) as described previously^60,61^. Briefly, after overnight growth, U2Os cells expressing the P2Y_1_ receptor were first treated with an antagonist for 20 min before the treatment with agonist and incubated for another 60 min. IP-1 detection reagents were added as instructed by the manual from the manufacturer. The assay plates were read on a Mithras LB940 reader (Berthold Technologies, Oak Ridge, TN) using a time-resolved fluorescence ratio (665/620 nm).

#### cAMP assay

U2Os cells expressing the P2Y_12_ receptor were cultured in DMEM medium containing 10% fetal bovine serum, 100 units/ml penicillin, 100 µg/ml streptomycin and 2 µmol/ml glutamine. For the assay of cAMP accumulation, cells were plated in 96-well plates in 100 µl medium overnight. Cells were then treated with assay buffer containing rolipram (10 mM) and antagonists for 20 min followed by the addition of agonists and incubate for 10 min. After 10 min incubation with agonist, Forskolin (10 μM) was added to the mixture and the incubation was continued for another 10 min. The reaction was terminated upon removal of the supernatant and addition of 100 µl Tween-20 (0.3%). Intracellular cAMP levels were measured with an ALPHAScreen cAMP assay kit as instructed by the manufacturer (PerkinElmer). Binding and functional parameters were calculated using Prism 7.0 software (GraphPAD, San Diego, CA, USA). Data were expressed as mean ± sem.

#### FLIPR assay

For agonist assay: cell line expressing target receptor was seeded in a 384-well black-wall, clear-bottom plate about 18 hours prior to the day of experiment and maintained at 37 °C/5% CO_2_. Then, 20 μl of dye-loading solution was added into the wells and the plates were subsequently placed into a 37 °C incubator for 60 min, followed by a 15 min incubation at room temperature. At last, 10 μl of compounds or control agonist were added into respective wells of the assay plate during reading in FLIPR.

For antagonist assay: cell line expressing target receptor was seeded in a 384-well black-wall, clear-bottom plate about 18 hours prior to the day of experiment and maintained at 37 °C/5% CO_2_. Then the dye-loading solution and 10 μl of control antagonist and test samples were added into respectively wells in sequence. The plates were subsequently placed into a 37 °C incubator for 60 min, followed by a 15 min incubation at room temperature. At last, 12.5 μl of control agonist were added into respective wells of the assay plate during reading in FLIPR.

## Electronic supplementary material

Supplementary Information
