# Author Correction: Salvianolic acids from antithrombotic Traditional Chinese Medicine Danshen are antagonists of human P2Y_1_ and P2Y_12_ receptors

**DOI:** 10.1038/s41598-018-29406-6

**Published:** 2018-07-24

**Authors:** Xuyang Liu, Zhan-Guo Gao, Yiran Wu, Raymond C. Stevens, Kenneth A. Jacobson, Suwen Zhao

**Affiliations:** 1 iHuman Institute, ShanghaiTech University, Shanghai, 201210 China; 2 School of Life Science and Technology, ShanghaiTech University, Shanghai, 201210 China; 3 Key Laboratory of Computational Biology, CAS-MPG Partner Institute for Computational Biology, Shanghai Institutes for Biological Sciences, Chinese Academy of Sciences, Shanghai, 20031 China; 4 University of Chinese Academy of Sciences, No. 19A, Yuquan Road, Beijing, 100049 China; 5 Molecular Recognition Section, Laboratory of Bioorganic Chemistry, National Institute of Diabetes and Digestive and Kidney Diseases, National Institutes of Health, Bethesda, Maryland 20892 USA

Correction to: *Scientific Reports* 10.1038/s41598-018-26577-0, published online 24 May 2018

This Article and the accompanying Supplementary Information file contain errors.

In Figure 2, the wrong µM symbol was used. The correct Figure 2 appears below as Figure 1.

**Figure 1:**
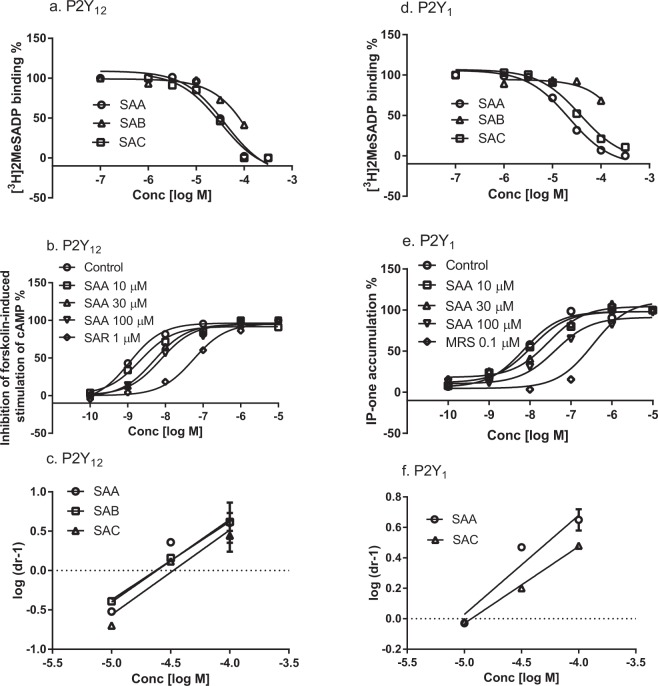
Radioligand binding assays and functional assays showed that SAA and SAC can bind and antagonize both P2Y_1_ and P2Y_12_ receptors, while SAB can bind and antagonize only the P2Y_12_ receptor. (**a**) Displacement curves of SAA, SAB and SAC against [^3^H]2MeSADP binding to the P2Y_12_ receptor. (**b**) Functional antagonism by SAA of 2MeSADP-induced inhibition of foskolin-stimulated cAMP accumulation in U2OS cells expressing the P2Y_12_ receptor. (**c**) Schild plots of the antagonism of SAA, SAB and SAC at the P2Y_12_ receptors. (**d**) Displacement curves of SAA, SAB and SAC against [^3^H]2MeSADP binding to the P2Y_1_ receptor. (**e**) 2MeSADPinduced IP-1 accumulation in U2OS cells expressing the P2Y_1_ receptor (compared to MRS2500). (**f**) Schild plots of antagonism by SAA and SAC at the P2Y_1_ receptor. Results are expressed as mean ± SEM. The K_i_ values from binding experiments and K_B_ values from Schild analyses of functional antagonism by SAA, SAB and SAC are listed in the text and are from at least three independent experiments. MRS, MRS2500; SAR, SAR216471.

In the Supplementary Information file, the title of Table S1 is incorrectly given as ‘SAA, SAB and SAC are not promiscuously binding compounds’. The correct Table S1 appears below as Table 1.

**Table 1:** Targets used in the off-target activities tested by the PDSP.

#	Protein name	#	Protein name
1	5-HT_1A_	24	D_3_
2	5-HT_1B_	25	D_4_
3	5-HT_1D_	26	D_5_
4	5-HT_1E_	27	GABA_A_
5	5-HT_2A_	28	H_1_
6	5-HT_2B_	29	H_2_
7	5-HT_2C_	30	H_3_
8	5-HT_3_	31	H_4_
9	5-HT_5A_	32	M_1_
10	5-HT_6_	33	M_2_
11	5-HT_7_	34	M_3_
12	α_1A_	35	M_4_
13	α_1B_	36	M_5_
14	α_1D_	37	δ-opioid
15	α_2A_	38	κ-opioid
16	α_2B_	39	μ-opioid
17	α_2C_	40	σ_1_
18	β_1_	41	σ_2_
19	β_2_	42	DAT
20	β_3_	43	NET
21	BZP rat brain site	44	SERT
22	D_1_	45	TSPO
23	D_2_		

Finally, in the Supplementary Information file, there is a typographical error in Figure S4 and its accompanying legend where ‘Epinenohrineon’ should read ‘Epinephrine’. The correct Figure S4 appears below as Figure 2.

**Figure 2:**
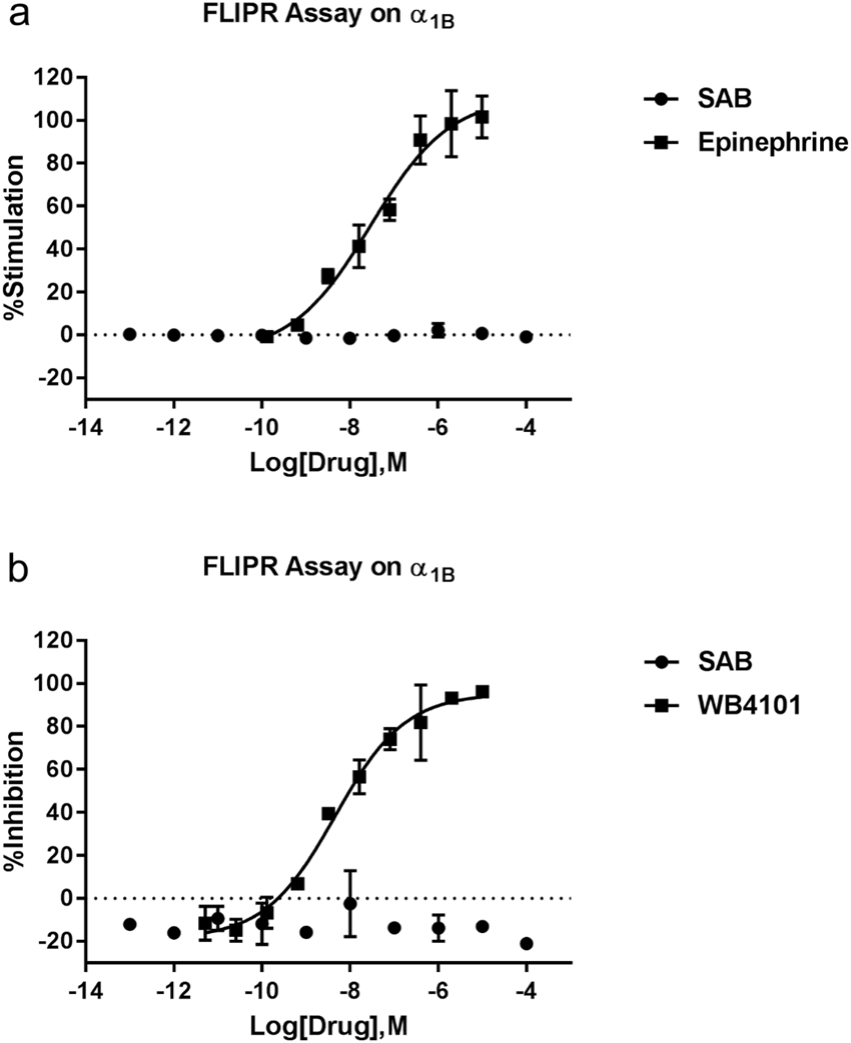
The functional activity of SAB on the adrenergic α_1B_ receptor were tested by FLIPR assays. SAB did not show agonist activity on α_1B_, epinephrine is a control agonist (EC50 29.8 nM) (**a**). SAB did not show antagonist activity on α_1B_, WB4104 is a control antagonist (IC50 4.51 nM) (**b**).

