# Supplementary material for: Salvianolic acids from antithrombotic Traditional Chinese Medicine Danshen are antagonists of human P2Y1 and P2Y12 receptors
Source: Sci Rep. 2018 May 24;8:8084. doi: 10.1038/s41598-018-26577-0 (PMC5967328; doi:10.1038/s41598-018-26577-0)
Supplement: Supplementary file 1 — Supplementary Information [file 41598_2018_26577_MOESM1_ESM.pdf]

Supplementary Information

Salvianolic acids from antithrombotic Traditional Chinese Medicine Danshen are antagonists of human P2Y$_1$ and P2Y$_{12}$ receptors

Xuyang Liu[1,2,3,4], Zhan-Guo Gao[5], Yiran Wu[1], Raymond C. Stevens[1], Kenneth A. Jacobson[5], Suwen Zhao[1,2,*]

[1]iHuman Institute, ShanghaiTech University, Shanghai 201210, China

[2]School of Life Science and Technology, ShanghaiTech University, Shanghai 201210, China

[3]Key Laboratory of Computational Biology, CAS-MPG Partner Institute for Computational Biology, Shanghai Institutes for Biological Sciences, Chinese Academy of Sciences, Shanghai, 20031, China

[4]University of Chinese Academy of Sciences, No. 19A, Yuquan Road, Beijing 100049, China

[5]Molecular Recognition Section, Laboratory of Bioorganic Chemistry, National Institute of Diabetes and Digestive and Kidney Diseases, National Institutes of Health, Bethesda, Maryland 20892, USA

[*]To whom correspondence should be addressed. Email: zhaosw@shanghaitech.edu.cn

Table S1. SAA, SAB and SAC are not promiscuously binding compounds

#	Protein name	#	Protein name
1	5-HT_{1A}	24	D_3
2	5-HT_{1B}	25	D_4
3	5-HT_{1D}	26	D_5
4	5-HT_{1E}	27	$GABA_A$
5	5-HT_{2A}	28	H_1
6	5-HT_{2B}	29	H_2
7	5-HT_{2C}	30	H_3
8	5-HT_3	31	H_4
9	5-HT_{5A}	32	M_1
10	5-HT_6	33	M_2
11	5-HT_7	34	M_3
12	α_{1A}	35	M_4
13	α_{1B}	36	M5
14	α_{1D}	37	δ-opioid
15	α_{2A}	38	κ-opioid
16	α_{2B}	39	μ-opioid
17	α_{2C}	40	σ_1
18	β_1	41	σ_2
19	β_2	42	DAT
20	β_3	43	NET
21	BZP rat brain site	44	SERT
22	D_1	45	TSPO
23	D_2		

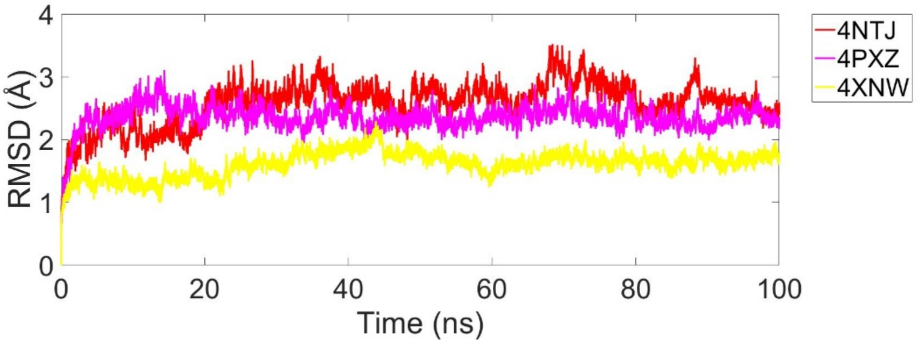

Figure S1. RMSD of Cα atoms compared with initial state in the MD simulations. The MD simulations were started from three different PDBs: 4NTJ ($P2Y_{12}$ receptor), 4PXZ ($P2Y_{12}$ receptor), and 4XNW ($P2Y_1$ receptor).

a

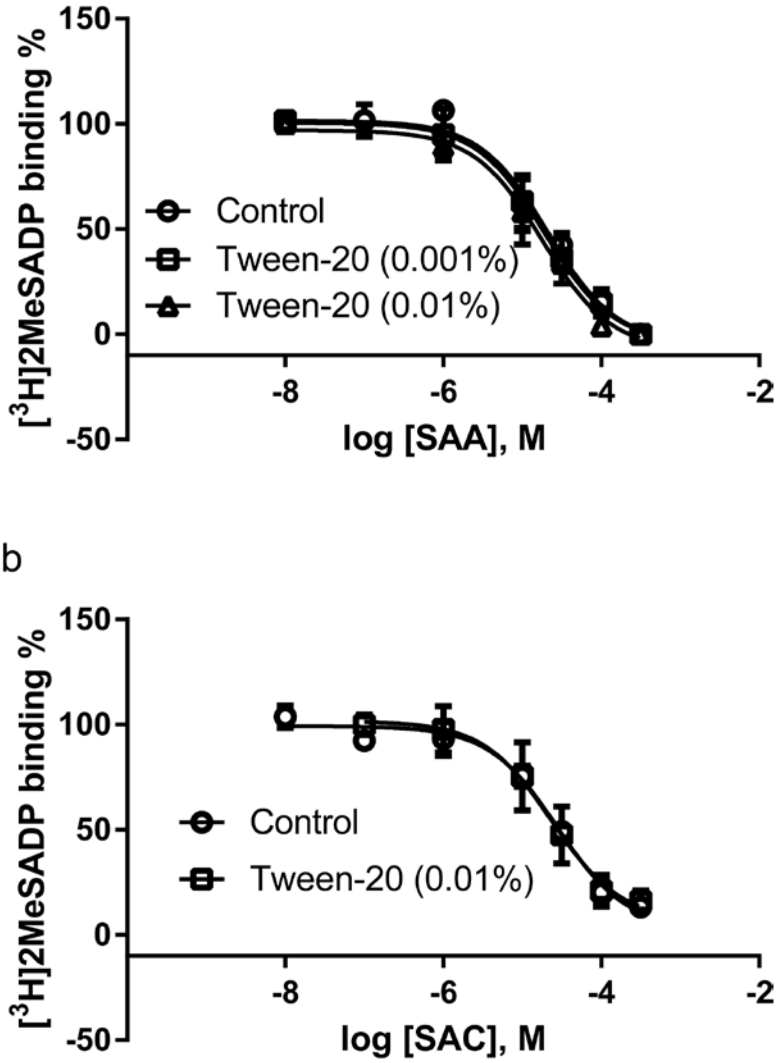

Figure S2. The effects of Tween-20 on the inhibition by SAA (a) and SAC (b) of [^{3}H]2MeSADP binding to the human $P2Y_1$ receptor. Data are expressed Mean ± SD from 2-3 independent experiments. The average IC_{50} values of SAA in the absence and presence of 0.01% Tween-20 are 21.2 and 19.3 μM, respectively. The IC_{50} values of SAC in the absence and presence of 0.01% Tween-20 are 29.4 and 23.7 μM, respectively. The critical micelle concentration (CMC) of Tween-20 is 0.0074%.

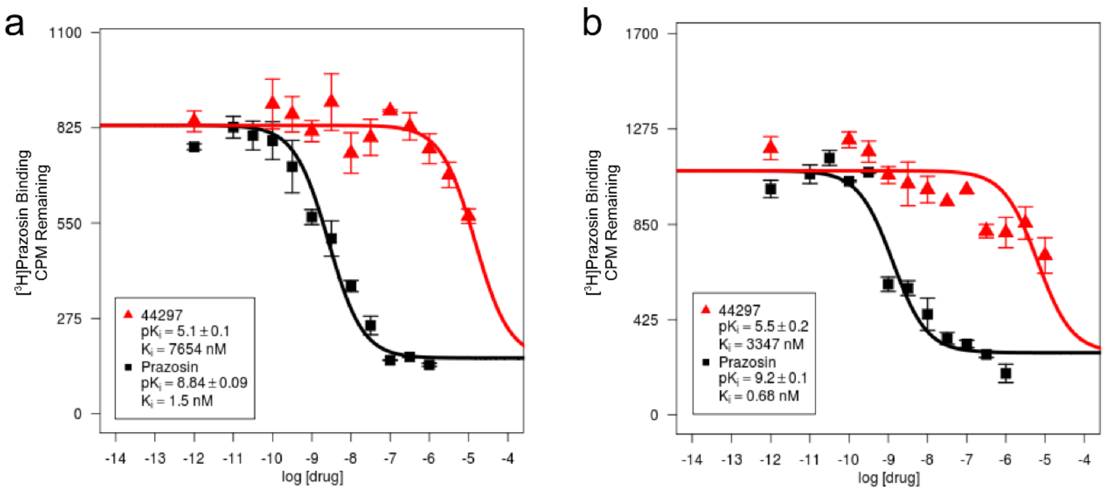

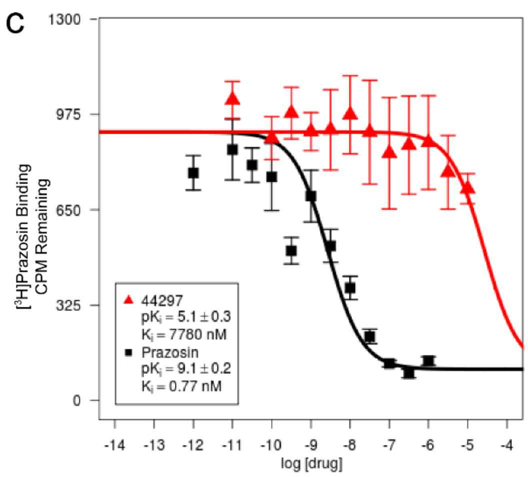

d

	1	2	3	Average
K_i (nM)	7654	3347	7780	6260

Figure S3. Binding activity of SAB (compound 44297) to the α_{1B} adrenergic receptor was measured by [^{3}H]Prazosin binding assays for three repeats (a-c). Prazosin is an inverse agonist of the α_{1B} adrenergic receptor. The average K_i of SAB was determined to be 6.26 μM (d).

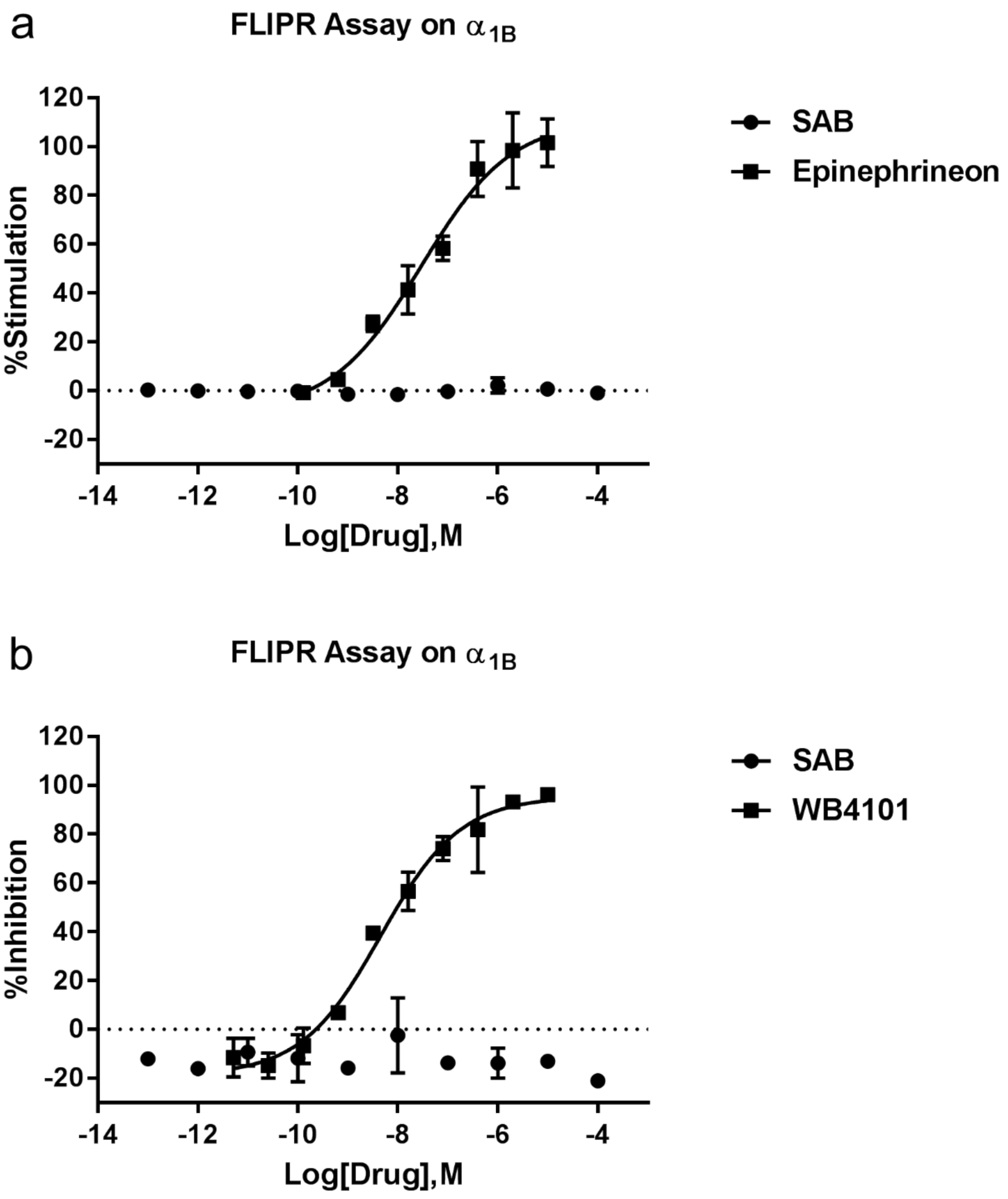

Figure S4. The functional activity of SAB on the adrenergic α_{1B} receptor were tested by FLIPR assays. SAB did not show agonist activity on α_{1B}, epinephrineon is a control agonist (EC50 29.8 nM) (a). SAB did not show antagonist activity on α_{1B}, WB4104 is a control antagonist (IC50 4.51nM) (b).
